# Exploring psychological distress among psychiatric nurses in Tunisia

**DOI:** 10.1192/j.eurpsy.2024.1256

**Published:** 2024-08-27

**Authors:** H. Khiari, A. Hakiri, M. Bouchendira, R. Ghachem

**Affiliations:** ^1^Psychiatry B; ^2^Psychiatry G, Razi hospital, Mannouba, Tunisia

## Abstract

**Introduction:**

Nurses working in psychiatric departments regularly encounter intricate, stress-inducing, and emotionally challenging situations. The mental well-being of these nurses directly influences the quality of care they deliver.

**Objectives:**

To assess the prevalence of psychological distress among psychiatric nurses and to identify the socio-demographic and clinical factors associated with it.

**Methods:**

Cross-sectional, descriptive, and analytical study conducted over the course of one month from October 11^th^ to November 8^th^ 2023. Participants included were psychiatric nurses working in Razi Hospital, Tunisia. We collected data using pre-established questionnaire which included socio-demographic and clinical data of the participants. The assessment of psychological distress was conducted using the Depression, Anxiety and Stress Scale (DASS-21), validated in Arabic. Statistical analysis was performed using the Statistical Package for Social Sciences (SPSS) in its 25th version

**Results:**

We collected data from 55 nurses working in Razi psychiatry hospital during the time of the study. Among them, 80% (n=44) were female. Their median age was 35 (Min=25, Max=62). Most of participants were married (81.8%, n=45) and 70.9 (n=39) had kids. In our sample, 5.5% (n=3) and 23.6% (n=13) had respectively personal psychiatric and somatic history. Some addictive behaviors were identified among our participants, especially smoking (14.5%, n=379) and alcohol use (3.6%, n=2).

Regarding working conditions, 81.8% (n=45) were assigned shift work. They worked in the men’s ward (43.6%, n=24), the women’s ward (34.5%, n=19), or in both (21.8%, n=12). Furthermore, 45.5% (n=25) reported witnessing a suicide attempt during their work, and 74.5% (n=41) were victims of aggression, primarily by patients (82.5%, n=33). Sixty percent (n=33) said expressed a desire to transfer.

Moderate to extremely severe depression, anxiety and stress was observed in respectively 34.5% (n=19), 61.8% (n=34) and 36.4% (n=20) of cases.

A significant association was found between stress among psychiatry nurses and personal somatic history (p <10^-3^). No further links were found between depression, anxiety, stress and other clinical factors.

**Conclusions:**

These results emphasize the difficult working environment within psychiatric settings, emphasizing the critical requirement for specific interventions aimed at improving the mental health and well-being of psychiatric nurses.

**Disclosure of Interest:**

None Declared

